# Oscillatory Motion of a Camphor Disk on a Water Phase with an Ionic Liquid Sensitive to Transition Metal Ions

**DOI:** 10.1021/acs.jpcb.4c07310

**Published:** 2024-12-26

**Authors:** Hua Er, Yukang Bai, Muneyuki Matsuo, Satoshi Nakata

**Affiliations:** † School of Chemistry and Chemical Engineering, Ningxia Key Laboratory of Solar Chemical Conversion Technology, Key Laboratory for Chemical Engineering and Technology, State Ethnic Affairs Commission, North Minzu University, Yinchuan 750021, P. R. China; ‡ Graduate School of Integrated Sciences for Life, Hiroshima University, 1-3-1 Kagamiyama, Higashi-Hiroshima 739-8526, Hiroshima, Japan; § Graduate School of Arts and Sciences, The University of Tokyo, 3-8-1 Komaba, Meguro, Tokyo 153-8902, Japan

## Abstract

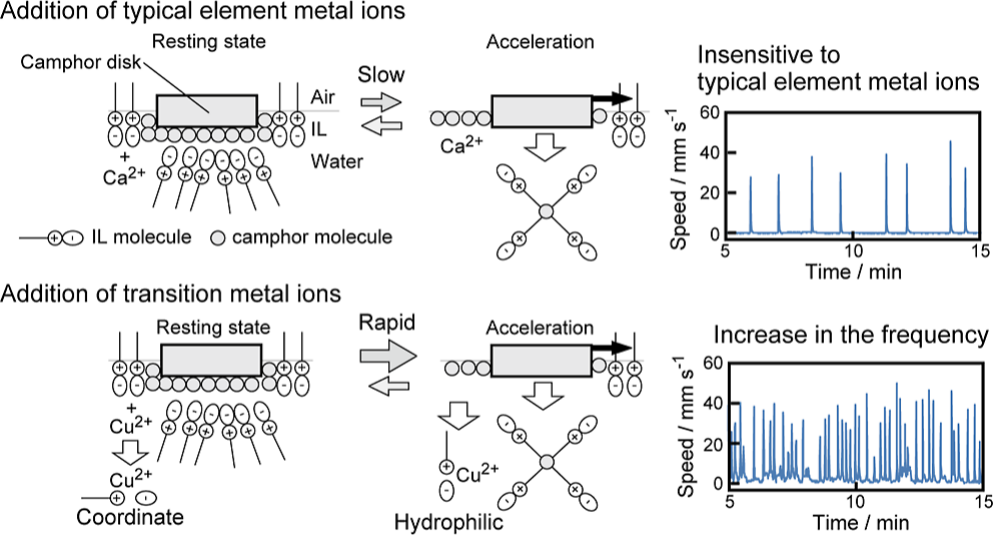

We investigated oscillatory motion of a camphor disk floating on water containing 5 mM hexylethylenediaminium trifluoroacetate (HHexen-TFA) as an ionic liquid (IL). The frequency of the oscillatory motion increased with increasing concentrations of the transition metal ions Cu^2+^ and Ni^2+^ but was insensitive to Na^+^, Ca^2+^, and Mg^2+^, the typical metal ions in the water phase. The surface tension of the water phase containing 5 mM HHexen-TFA also increased with increasing concentrations of Cu^2+^ and Ni^2+^ but was insensitive to Na^+^, Ca^2+^, and Mg^2+^. Based on density functional theory, metal-ion species-dependent frequency response is discussed with regard to surface tension as the force of self-propulsion and complex formation between HHexen-TFA and metal ions. These results suggest that complex formation between the transition metal ions (Cu^2+^, Ni^2+^) and the ethylenediamine group in the IL increases the surface tension around the camphor disk, resulting in an increase in the frequency of oscillatory motion with increasing concentrations of Cu^2+^ or Ni^2+^. The present study suggests that the nature of self-propulsion can be created by complexation, which changes the force of self-propulsion.

## 1 Introduction

Development of inanimate self-propelled objects has been investigated to carry matter in a micrometer or millimeter space.^1−6^ Self-propelled objects are mainly classified into two types based on the driving force of their motion. One driving force is electrophoresis or bubbles produced on noble metals, such as Pt nanorods and Au Janus particles.^7−16^ The other is the spatial gradient in interfacial tension around an amphiphilic object.^17−25^ Most inanimate self-propelled objects move randomly or unidirectionally depending on the intrinsic or extrinsic cause, and the direction and speed of motion are determined by the inhomogeneity of the internal or external field, for example, the shape of the object or the electromagnetic field.^11,25−28^ In contrast, animate self-propulsion, such as bacterial motion, can characteristically change the nature of motion while responding to their physicochemical environments.^29^ The introduction of nonlinear properties, such as oscillation and pattern formation, into inanimate self-propelled systems is one strategy to enhance the autonomy of the systems because characteristic features of motion can be created based on physicochemical nonlinearity.^22−25,30−33^

On the other hand, ionic liquids (ILs), composed of both cationic and anionic parts, have been studied as electrolytes or solvents.^34,35^ ILs can potentially introduce physicochemically controllable nonlinearity into inanimate self-propulsion owing to their characteristic solubility and cation-sensitive coordination ability, which depend on their polar groups and alkyl chain length.^35−39^ We previously reported a self-propelled camphor pill or boat on water or surfactant aqueous solutions such as sodium dodecyl sulfate (SDS).^25,32^ Recently, we found that the nature of camphor disk motion could be changed by the addition of the ionic liquids hexylethylenediaminium trifluoroacetate (HHexen-TFA) and hexylammonium trifluoroacetate (HHexam-TFA) in water.^40^ That is, bifurcation of self-propulsion among uniform motion, repetition between rest and motion, and no motion was observed depending on the IL concentration.

**Scheme 1:**
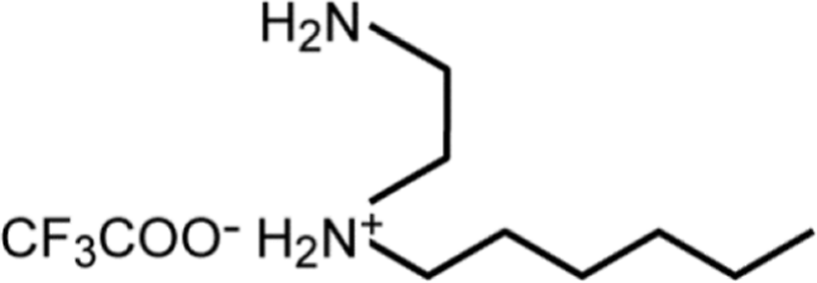
Chemical Structure of the Ionic Liquid HHexen-TFA Used in the Present Article

In this study, a camphor disk exhibited oscillatory motion between rest and motion on an aqueous solution of HHexen-TFA (see Scheme 1). The features of the oscillatory motion were sensitive to transition metal ions; that is, the frequency of the oscillatory motion increased with increasing concentrations of Cu^2+^ and Ni^2+^ added to the IL aqueous phase. In contrast, the frequency of the oscillatory motion was not sensitive to Na^+^, Ca^2+^, and Mg^2+^, which are typical metal ions. Different responses in the frequency of the oscillatory motion depending on the metal ion species are discussed regarding the surface tension and complex formation between HHexen-TFA and the metal ion species. The present study suggests that the features of the oscillatory motion can be altered by the interfacial properties of the complex formed with the IL, depending on the metal ion species.

## 2 Experimental Section

The purity of HHexen-TFA (C_10_H_21_N_2_O_2_F_3_), as illustrated in Scheme 1,^37,38^ was evaluated using ^13^C NMR measurement on Bruker 400 MHz spectrometer and CHN elemental analysis on GmbH Vario EL instrument. (+)-Camphor (C_10_H_16_O, with a purity >96%) was supplied by Shanghai Macklin. A camphor disk characterized by a diameter of 6.0 mm, thickness of 1.0 mm, and a mass of approximately 30 mg, was prepared according to the previous study.^25,32^ The disk was delicately positioned to float on a 40 mL, 4 mm deep aqueous solution containing HHexen-TFA, which was contained within a glass Petri dish featuring an inner diameter of 120 mm and a depth of 15 mm. Ultrapure water was produced on a water purification machine manufactured by Ningbo Dansboton Environmental Protection Tech. Co., Ltd., China. The experiments were performed at least three times under each experimental condition to ascertain the reproducibility of obtained phenomena. The motion of the camphor disk was meticulously observed and recorded using an Olympus STYLUS XZ-2 model digital video camera (minimum time resolution: 1/30 s) in an air-conditioned room maintained at 298 ± 2 K. The self-propelled movement of the object was subsequently analyzed using ImageJ software, provided by the National Institutes of Health in the United States. Additionally, the surface tension at the air/aqueous interface of an aqueous solution containing HHexen-TFA was precisely measured using a BZY-2 model surface tensiometer manufactured by Shanghai Heng Ping Instrument Factory.

The intermolecular interaction energy between the IL and metal chloride (molar ratio: 1:1) was numerically calculated based on density functional theory (DFT) with M06-2X functional and 6-311G(d,p) basis set using the Gaussian 09 W software package.^41^ The optimized configurations of the IL–metal chlorides (CuCl_2_, NiCl_2_, MgCl_2_, and CaCl_2_) are described in the Supporting Information.

## 3 Results

**Figure 1:**
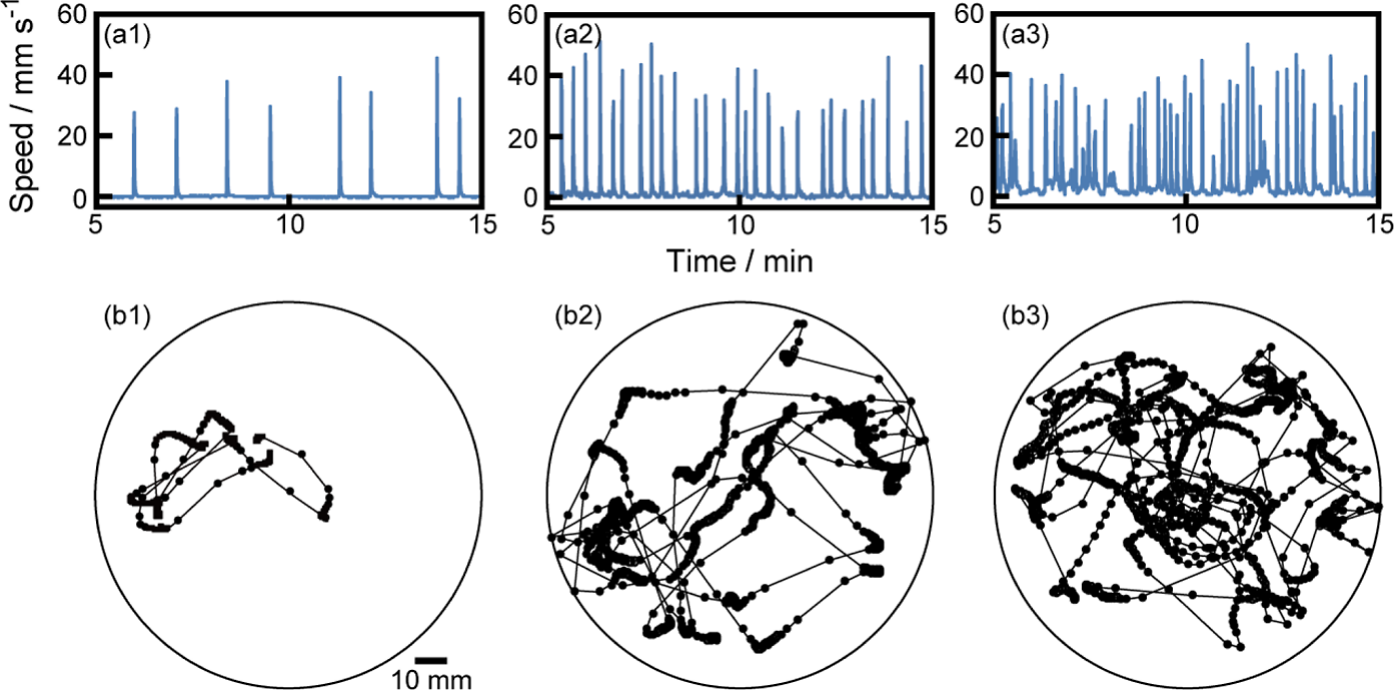
(a) Temporal change of the speed for camphor motion and (b) trajectories of the center position of a camphor disk on 5 mM HHexen-TFA aqueous solution for different concentrations of CuCl_2_ [(1) 0, (2) 2, and (3) 5 mM] from *t* = 5 to 15 min (top view). The time interval of motion was 1/30 s. The circle in (b) corresponds to the Petri dish. The movies of motion in (1), (2), and (3) are provided in the Supporting Information as Movies S1, S2, and S3, respectively.

First, we monitored the self-propulsion of a camphor disk on a 5 mM HHexen-TFA aqueous phase at different concentrations of CuCl_2_, as shown in Figure 1. Oscillatory motion between rest and motion was observed for 5 mM HHexen-TFA in the absence of CuCl_2_ [Figure 1(1)]. The frequency of the oscillatory motion increased with increasing concentration of CuCl_2_ [Figure 1(2),(3)]. The trajectories of the oscillatory motion were random for 0, 2, and 5 mM CuCl_2_. The maximum speed of the oscillatory motion was almost independent of the concentration of CuCl_2_. The variations in the speed and trajectory of the oscillatory motion of 5 mM HHexen-TFA with the addition of CaCl_2_ are shown in Figure S1 of the Supporting Information.

**Figure 2:**
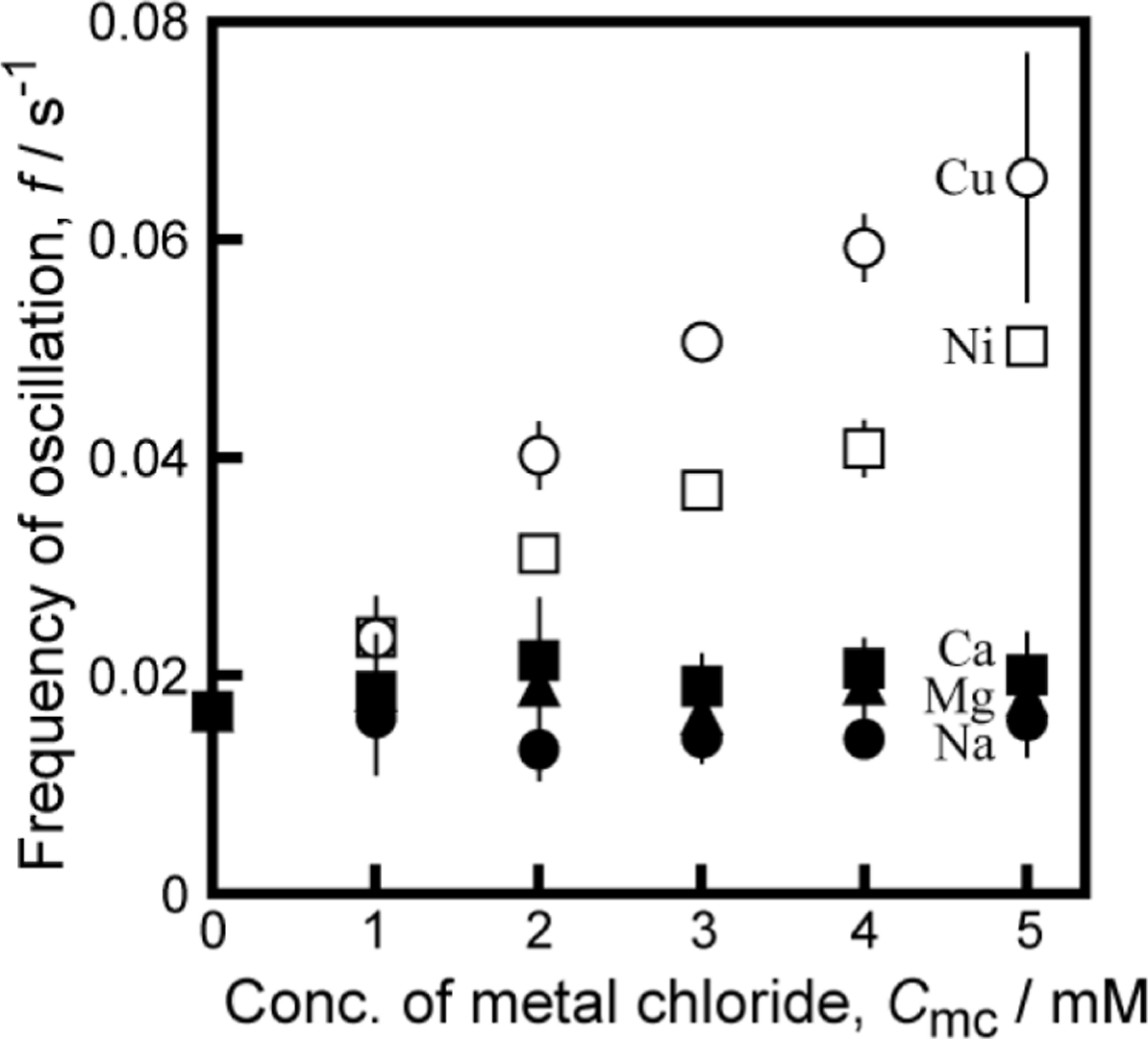
Frequency of oscillatory motion depending on the concentration of metal chlorides (NaCl, CaCl_2_, MgCl_2_, CuCl_2_, NiCl_2_), *C*_mc_, in a 5 mM HHexen-TFA aqueous solution. Error bars represent standard deviation.

Figure 2 shows the frequency of the oscillatory motion of a camphor disk depending on the concentration of the metal chlorides, CuCl_2_ (empty circles), NiCl_2_ (empty squares), CaCl_2_ (filled squares), MgCl_2_ (filled triangles), and NaCl (filled circles), *C*_mc_, in a 5 mM HHexen-TFA aqueous solution. The frequency of the oscillatory motion increased with increasing concentrations of CuCl_2_ and NiCl_2_. In contrast, the frequency did not change with the concentrations of CaCl_2_, MgCl_2_, or NaCl. The amplitude of the oscillatory motion was almost independent of the metal chloride concentration used in this study (see Figure S2 on the amplitude of oscillatory motion in the Supporting Information).

**Figure 3:**
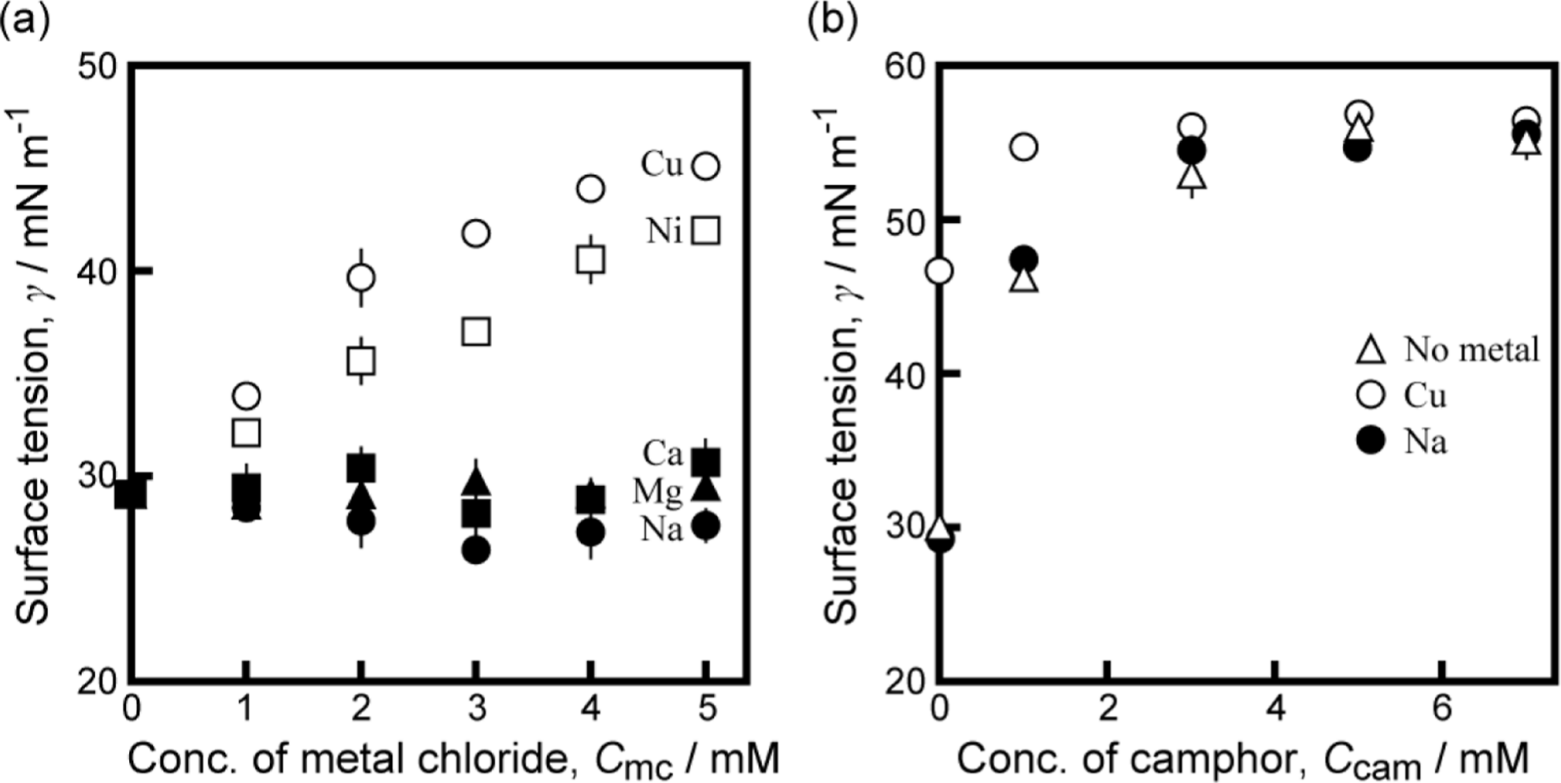
Surface tension depending on the concentration of (a) metal chloride, NaCl (filled circles), CaCl_2_ (filled squares), MgCl_2_ (filled triangles), CuCl_2_ (empty circles), NiCl_2_ (empty squares)), *C*_mc_, in a 5 mM HHexen-TFA aqueous solution and (b) camphor, *C*_cam_, in a 5 mM HHexen-TFA aqueous solution with 5 mM metal chloride (NaCl (filled circles), CuCl_2_ (empty circles) or without metal chloride (empty triangles). Error bars represent standard deviation.

Measurement of the surface tension of the aqueous phase is significant to elucidate the mechanism by which the frequency of oscillatory motion is sensitive to CuCl_2_ and NiCl_2_ because the force of camphor self-propulsion depends on the spatial difference in the surface tension around it on the aqueous surface.^25^Figure 3a shows the surface tension (γ) of the 5 mM HHexen-TFA aqueous phase depending on *C*_mc_ for CuCl_2_, NiCl_2_, CaCl_2_, MgCl_2_, and NaCl. γ increased with increasing concentrations of CuCl_2_ and NiCl_2_. In contrast, γ was not sensitive to CaCl_2_, MgCl_2_, and NaCl. Figure 3b shows γ of the 5 mM HHexen-TFA aqueous phase with or without 5 mM CuCl_2_ or NaCl, depending on the concentration of camphor, *C*_cam_. γ was increased with *C*_cam_. γ for CuCl_2_ was higher than that for NaCl or without metal chloride at *C*_cam_ ≤ 3 mM, but was similar at *C*_cam_ ≥ 5 mM.

To investigate the coordination of metal ions with the ethylenediamine group and TFA in the IL, we calculated the interaction energies between the IL and metal chlorides. The configurations of one ethylenediamine in IL–one metal chloride (MgCl_2_, CaCl_2_, NiCl_2_, or CuCl_2_) complex were individually optimized at the M06-2X/6-311G(d,p) level using DFT calculations, as illustrated in Figures S3 of the Supporting Information. The basis set superposition error (BSSE) and zero-point vibrational energy (ZPVE)-corrected intermolecular interaction energies (Δ*E*_0_^BSSE^/kJ mol^–1^) for the optimized configurations involving coordination with ethylenediaminium in the IL for different metal ions are presented in Table 1. The Δ*E*_0_^BSSE^ for Ni^2+^ and Cu^2+^ were more negative than those for Mg^2+^ and Ca^2+^. The configurations of one TFA in IL–one metal chloride (MgCl_2_, CaCl_2_, NiCl_2_, or CuCl_2_) complex are shown in Figure S4 of the Supporting Information. Regarding the stability of the coordination with TFA and metal ions, similar values of Δ*E*_0_^BSSE^ were obtained for Mg^2+^, Ca^2+^, Ni^2+^, and Cu^2+^ (see the right side of Table 1).

**Table 1:** Intermolecular Interaction Energy, Δ*E*_0_^BSSE^ (kJ mol^–1^), of the Optimized Configurations for the IL-Metal Chloride (Coordinated with Cation Ethylenediamine (Left Side) and Anion TFA (Right Side))

metal ions	Δ*E*_0_^BSSE^/kJ mol^–1^ (coordinated with cation ethylenediamine)	Δ*E*_0_^BSSE^/kJ mol^–1^(coordinated with anion TFA)
Mg^2+^	–28	–41
Ca^2+^	–26	–41
Ni^2+^	–42	–38
Cu^2+^	–43	–39

## 4 Discussion

Based on the present results and previous works,^22−25,34−40,42−44^ we discuss the increase in the frequency of the oscillatory motion of a camphor disk floating in a 5 mM HHexen-TFA aqueous phase with the addition of CuCl_2_ and NiCl_2_. The force of camphor self-propulsion is due to the surface tension difference around the disk as a one-dimensional system; that is, Δγ = γ_cL_ – γ_cR_, where γ_cL_ and γ_cR_ are the surface tensions on the left and right edges of the camphor disk, respectively.^25^ On the water phase without HHexen-TFA and metal ions, the camphor disk continuously supplies camphor molecules onto the water surface, resulting in continuous motion. If the camphor disk moves continuously, the surface tension difference remains due to the sublimation and dissolution of camphor molecules from the water surface into the air and water phase, respectively, and continuous motion is maintained.^25^

**Figure 4:**
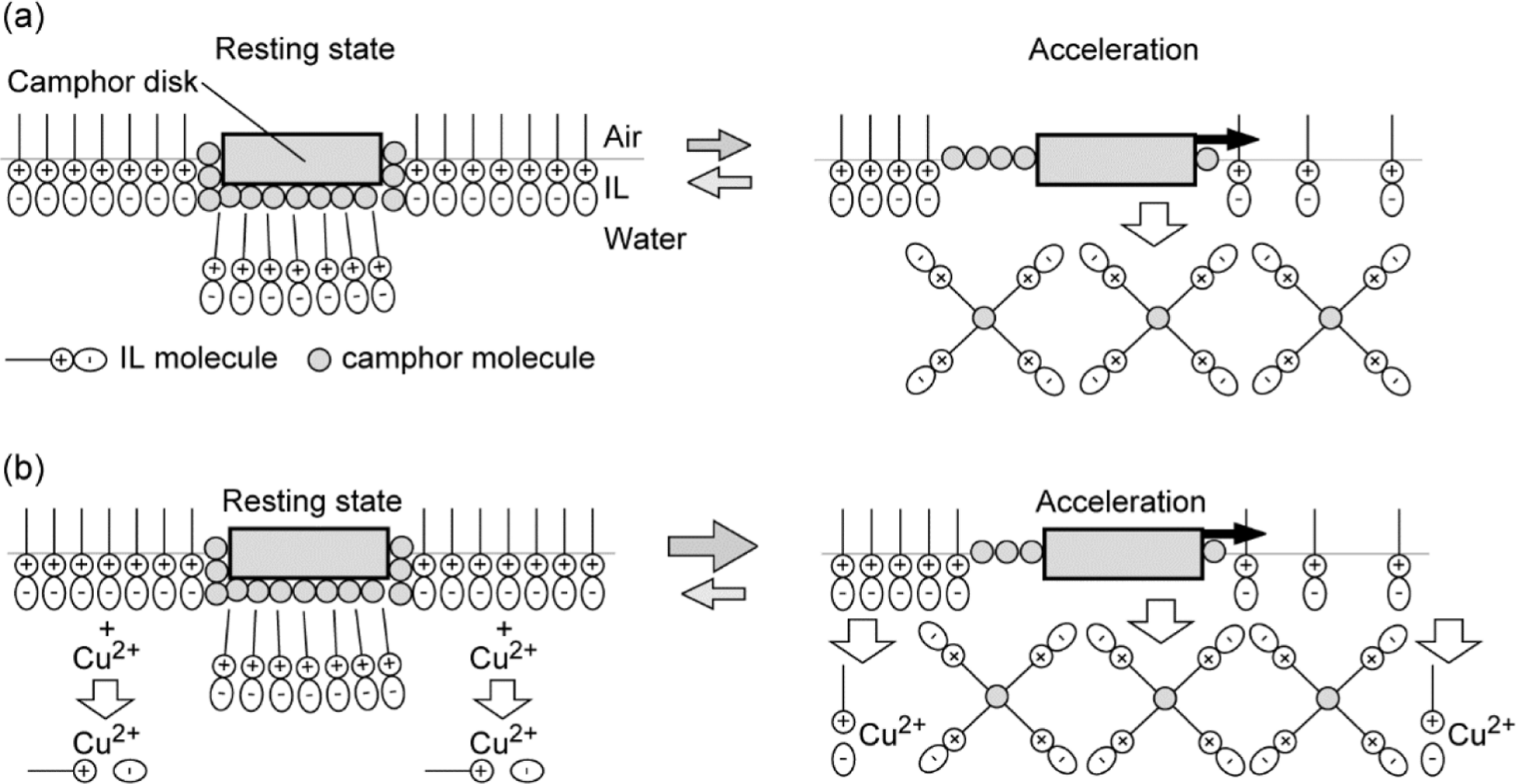
Schematic representation of the mechanism of oscillatory motion of a camphor disk placed on an HHexen-TFA aqueous phase: (a) without metal chloride and (b) with CuCl_2_ (side view).

In contrast, the oscillatory motion observed for 5 mM HHexen-TFA (see Figures 1 and S1) suggests that the camphor disk alternately gains and loses the force of self-propulsion, as schematically indicated in Figure 4. We previously reported the mechanism of the oscillatory motion of the camphor disk in 5 mM HHexen-TFA.^40^ The surface tension γ for the saturated aqueous solution of camphor (∼55 mN m^–1^ at ∼7 mM) is higher than that for 5 mM HHexen-TFA without camphor (∼30 mN m^–1^). Therefore, the camphor disk remains at rest because the water surface is covered by a layer of HHexen-TFA molecules (see the left side of Figure 4a). However, the surface tension is increased by the addition of camphor (see Figure 3b) since camphor molecules are distributed on the water surface in the place of HHexen-TFA, and the mixture of HHexen-TFA and camphor is dissolved into the water phase. As a result, the camphor disk can accelerate toward regions of lower HHexen-TFA concentration, that is, higher surface tension (right side of Figure 4a). When the camphor disk moves to another location with a higher HHexen-TFA concentration, it stops again because the state around the camphor disk returns to the resting state. Thus, the oscillatory motion is repeated between resting at regions of higher HHexen-TFA concentration and acceleration due to surface tension changes resulting from the mixture of HHexen-TFA and camphor (Figure 4a).

Figure 3a suggests that the HHexen-TFA molecular layer is desorbed from the air/water interface into the bulk phase with the addition of CuCl_2_ or NiCl_2_, as indicated at the left side of Figure 4b. This occurs because HHexen-TFA coordinates with Cu^2+^ or Ni^2+^,^42−44^ and the water-soluble HHexen-TFA-Cu^2+^ or HHexen-TFA-Ni^2+^ complex dissolve into the water phase. Table 1 suggests that the coordination of the ethylenediamine group in HHexen-TFA to transition metal ions (Ni^2+^ and Cu^2+^) has an important role in the frequency of the oscillatory motion. As the resting time decreases, the frequency of the oscillatory motion increases with increasing concentration of CuCl_2_ or NiCl_2_. In contrast, the frequency of the oscillatory motion is independent of the addition of NaCl, CaCl_2_, and MgCl_2_ because the surface tension is independent of their concentrations (see Figure 3a). Moreover, the surface tension dependence on *C*_cam_ without metal chloride is similar to that for 5 mM NaCl (see Figure 3b). These results suggest that it is difficult to form water-soluble complexes with Na^+^, Ca^2+^, or Mg^2+^.

The differences in the frequency responses of the oscillatory motion between the transition metal ions (Cu^2+^, Ni^2+^) and the typical metal ions (Na^+^, Ca^2+^, and Mg^2+^) (see Figures 1, S1, S2, and 2) may be due to the stability of the complex between HHexen-TFA and the cation species, especially the stability of the complex between the ethylenediamine group and Cu^2+^ or Ni^2+^. This is consistent with the fact that the stability constants (log *K*) for the transition metal ions are higher than those of typical main group metal ions—for example, for ethylenediamine:^45^ Ca^2+^, 0.20; Mg^2+^, 0.37; Ni^2+^, 6.98; and Cu^2+^, 10.06—which agrees with the experimental results in the present study.

Table 1 shows the differences in the HHexen-TFA complexes with various metal ions (Cu^2+^, Ni^2+^, Mg^2+^, and Ca^2+^), as verified by DFT calculations. The optimized structure of camphor coordinated with HHexen-TFA is shown in Figure S5 of the Supporting Information. That is, the coordination stabilities of the ethylenediamine group in HHexen-TFA with Cu^2+^ and Ni^2+^ (Δ*E*_0_^BSSE^ ∼ −42 kJ mol^–1^) are higher than those with Mg^2+^ and Ca^2+^(Δ*E*_0_^BSSE^ ∼ −27 kJ mol^–1^) (left side of Table 1). However, the coordination stabilities between TFA and the metal ions (Mg^2+^, Ca^2+^, Ni^2+^, and Cu^2+^) are similar (right side of Table 1). In other words, the coordination stability between the ethylenediamine group and Cu^2+^ or Ni^2+^ induces characteristic features of motion depending on the concentration.

## 5 Conclusions

In this study, we found oscillatory motion of a camphor disk which preferred dwelling at a certain location before switching its direction of motion, like run-and-tumble motion of bacteria. The frequency of the oscillatory motion of a camphor disk on water with HHexen-TFA was found to increase with the addition of Cu^2+^ and Ni^2+^ as transition metal ions but was insensitive to Na^+^, Ca^2+^, and Mg^2+^ as typical metal ions. We discussed the increase in the frequency of the oscillatory motion with increasing concentrations of Cu^2+^ and Ni^2+^ regarding the dependence of the surface tension on concentrations of metal chlorides and camphor. The solubility of the complex composed of HHexen-TFA and the divalent cations of transition elements, such as Cu^2+^ and Ni^2+^, plays an important role in increasing the frequency of the oscillatory motion. The effect of anions should be considered to understand the characteristic motion of a camphor disk on the ionic liquid aqueous phase with the addition of metal salt in the future work. The present study suggests that the inanimate nature of self-propulsion driven by the difference in surface tension can be designed by tuning the chemical and physical characteristics of the IL, such as its ability to form complexes with divalent cations. In other words, the metal cation species can be detected based on the features of the oscillatory motion of the camphor disk through coordination with the IL.

## Supporting Information Available

The detail of DFT calculation; Time-variation of the speed and trajectory of motion for a camphor disk on a 5 mM HHexen-TFA aqueous solution at 2 and 5 mM CaCl_2_; Amplitude of oscillatory motion of a camphor disk depending on the concentration of metal chloride in a 5 mM HHexen-TFA aqueous solution; Optimized configurations of ethylenediaminium in IL coordinated with metal chlorides; Optimized configurations of TFA in IL coordinated with metal chlorides; Optimized configuration of IL coordinated with camphor (PDF)
Movie S1 corresponds to Figure 1(1) (AVI)
Movie S2 corresponds to Figure 1(2) (AVI)
Movie S3 corresponds to Figure 1(3) (AVI)

## Supplementary Material

jp4c07310_si_001.pdf

jp4c07310_si_002.avi

jp4c07310_si_003.avi

jp4c07310_si_004.avi
