# Supplementary material for: Oscillatory Motion of a Camphor Disk on a Water Phase with an Ionic Liquid Sensitive to Transition Metal Ions
Source: J Phys Chem B. 2024 Dec 26;129(1):592–7. doi: 10.1021/acs.jpcb.4c07310 (PMC11726673; doi:10.1021/acs.jpcb.4c07310)
Supplement: Supplementary file 1 — jp4c07310_si_001.pdf [file jp4c07310_si_001.pdf]

Supporting Information (SI)

Oscillatory motion of a camphor disk on a water phase with an ionic liquid sensitive to transition metal ions

Hua Er[a,*], Yukang Bai[a], Muneyuki Matsuo[b,c], Satoshi Nakata[b,**]

[a] *School of Chemistry and Chemical Engineering, Ningxia Key Laboratory of Solar Chemical Conversion Technology, Key Laboratory for Chemical Engineering and Technology, State Ethnic Affairs Commission, North Minzu University, Yinchuan 750021, P. R. China*

[b] *Graduate School of Integrated Sciences for Life, Hiroshima University, 1-3-1 Kagamiyama, Higashi-Hiroshima, Hiroshima 739-8526, Japan*

[c] *Graduate School of Arts and Sciences, The University of Tokyo, 3-8-1 Komaba, Meguro, Tokyo 153-8902, Japan*

*To whom correspondence should be addressed. Tel.: +86-15709699185 E-mail: huaer0101@hotmail.com

**To whom correspondence should be addressed. Tel.: +81-82-424-7409, E-mail: nakatas@hiroshima-u.ac.jp

1. DFT calculation

The optimized 3D structures and interaction energies of IL (HHexen-TFA), metal chloride, and IL with metal chloride were computed using density functional theory at the M06-2X/6-311G(d,p) level. The interaction energy (ΔE_{int}) is defined as the difference between the energy of the molecule pair ($E_{molecule\ pair}$) and the energies of the individual molecules ($E_{molecules}$), as shown in equation S1. In this manuscript, the interaction energy is corrected for basis set superposition errors (BSSE) and zero-point vibrational energy (ZPVE), resulting in the corrected interaction energy ΔE_0^{BSSE}, which is explained further in equations S2-S3.

$$\Delta E_{int} = E_{molecules\ pair\ (IL\text{-}metal\ chloride)} - (E_{molecule\ (IL)} + E_{molecule\ (metal\ chloride)}) \quad (S1)$$

$$\Delta E_0 = \Delta E_{int} + \Delta ZPVE \quad (S2)$$

$$\Delta E_0^{BSSE} = \Delta E_0 + BSSE \quad (S3)$$

2. Self-propelled motion of a camphor disk on a 5 mM HHexen-TFA aqueous solution with the addition of $CaCl_2$

Figure S1 shows the self-propelled motion of a camphor disk on a 5 mM HHexen-TFA aqueous solution with the addition of $CaCl_2$. The frequency of oscillatory motion was slightly increased with an increase in the concentration of $CaCl_2$. The trajectory of oscillatory motion was random.

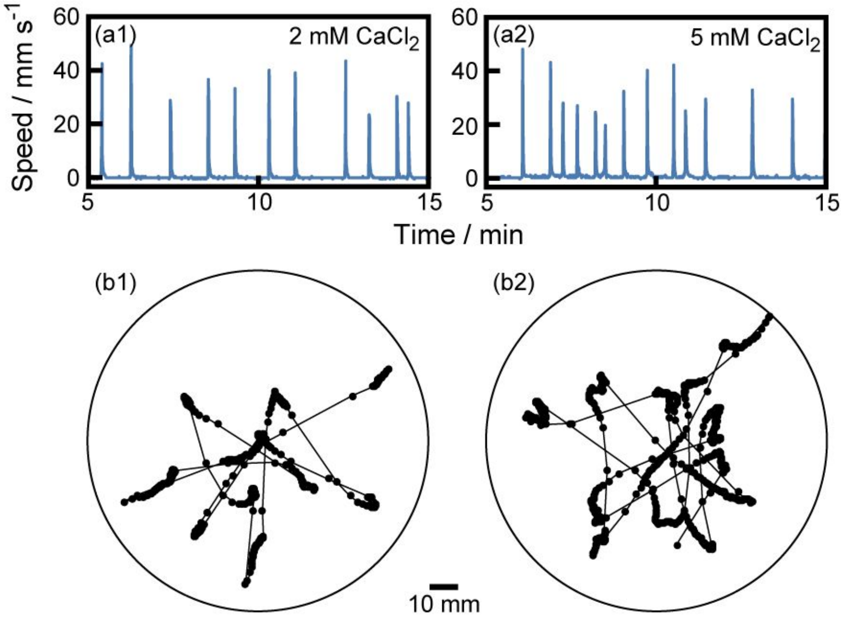

Figure S1. (a) Time-variation of the speed and (b) trajectory of motion for a camphor disk on a 5 mM HHexen-TFA aqueous solution with different concentrations of $CaCl_2$ ((1) 2 and (2) 5 mM).

3. Amplitude of oscillatory motion of a camphor disk depending on the concentration of metal chloride in a 5 mM HHexen-TFA aqueous solution

Figure S2 shows the amplitude of oscillatory motion of a camphor disk depending on the concentration of metal chloride, C, in a 5 mM HHexen-TFA aqueous solution. The amplitude of oscillatory motion of the camphor disk was almost independent of C for the metal chloride ($CuCl_2$, $NiCl_2$, $CaCl_2$, $MgCl_2$, and NaCl) in the 5 mM HHexen-TFA aqueous solution.

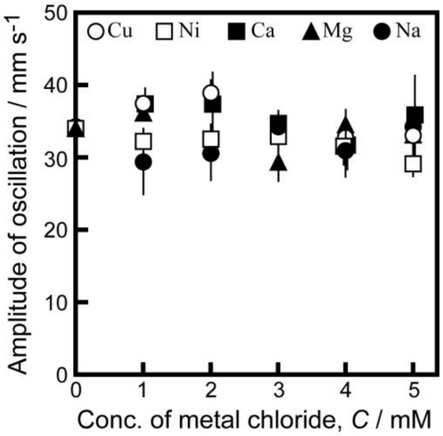

Figure S2. Amplitude of oscillatory motion of a camphor disk depending on the concentration of metal chloride (C) in a 5 mM HHexen-TFA aqueous solution.

4. Optimized configurations of ethylenediaminium in IL combined with metal chlorides

Figure S3 shows the optimized configurations of ethylenediaminium in IL and metal chlorides obtained from the DFT calculation to clarify the molecular interaction between them.

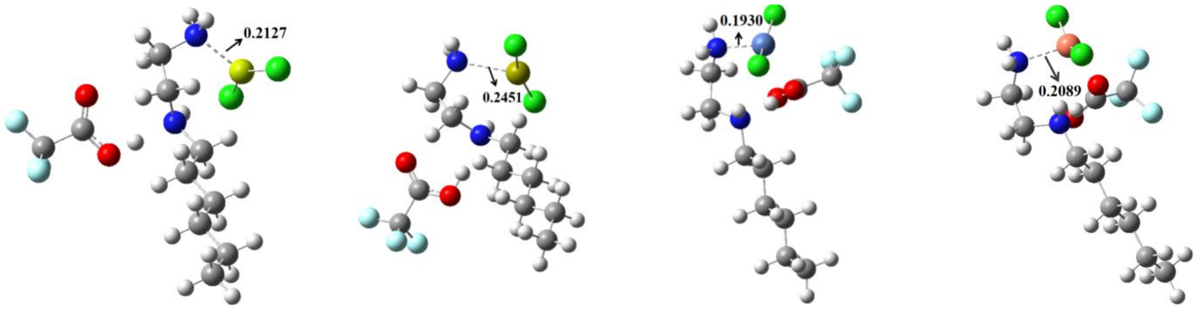

Mg^{2+} / ΔE_0^{BSSE} = -28 Ca^{2+} / ΔE_0^{BSSE} = -26 Ni^{2+} / ΔE_0^{BSSE} = -42 Cu^{2+} / ΔE_0^{BSSE} = -43

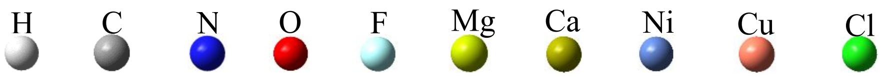

Figure S3. Metal chloride coordinated with ethylenediaminium in IL and the interaction energy (ΔE_0^{BSSE} / kJ·mol^{-1}). Individual numbers denote the length (nm) between NH_2 group and metal cation.

Figure S4 shows the optimized configurations of TFA in IL and metal chloride obtained from the DFT calculation to clarify the molecular interaction between them.

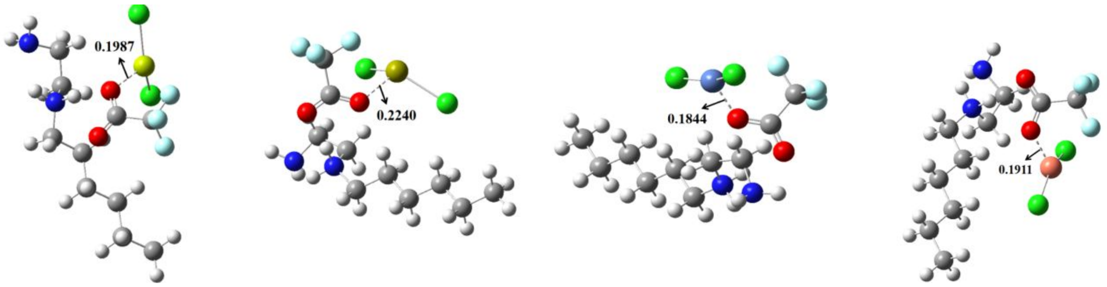

Mg^{2+} / ΔE_0^{BSSE} = -41 Ca^{2+} / ΔE_0^{BSSE} = -41 Ni^{2+} / ΔE_0^{BSSE} = -38 Cu^{2+} / ΔE_0^{BSSE} = -39

Figure S4. Metal chloride coordinated with TFA in IL and the interaction energy (ΔE_0^{BSSE} / kJ·mol^{-1}).

5. **Optimized configuration of IL combined with camphor**

Figure S5 shows the optimized configuration of IL combined with camphor obtained from the DFT calculation to clarify the molecular interaction between them. There is a hydrophobic interaction between camphor and alkyl chain in IL.

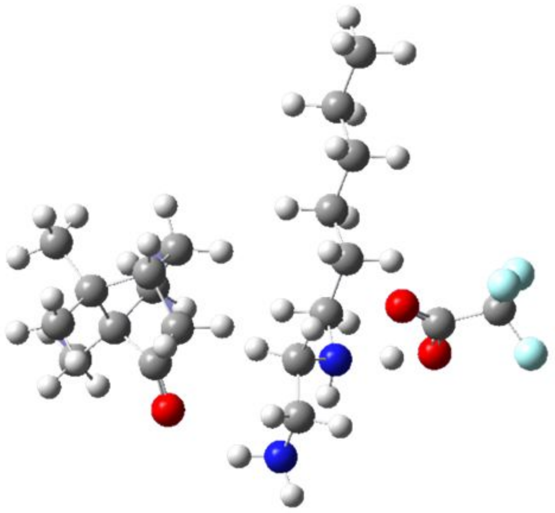

Figure S5. Optimized configuration of the camphor coordinated with alkyl chain in IL obtained from the DFT calculation.
